# Association of frailty index with incidence of chronic kidney disease: China Health and Retirement Longitudinal Study

**DOI:** 10.1007/s41999-024-01148-x

**Published:** 2025-01-15

**Authors:** Lisha Zhang, Yan Zhang, Yanru He, Fuxue Deng, Jiahong Xue

**Affiliations:** Department of Cardiovascular Medicine, The Second Affiliated Hospital, Xi’an Jiaotong University, Xi’an, 710004 Shaanxi People’s Republic of China

**Keywords:** Frailty index, Incidence of chronic kidney disease, Accumulation of frailty, Change of frailty status, Aging

## Abstract

**Aim:**

Frailty is an important risk factor for a wide range of chronic diseases and for mortality risk. This study aims to explore the relationship between frailty and incidence of chronic kidney disease (CKD), particularly on the change and accumulation of frailty.

**Methods:**

Frailty status was assessed using the frailty index (FI, constructed by 31 items) and categorized as robust, pre-frail, and frail. The accumulation and change in frailty were assessed on the basis of frailty status at baseline and a second survey 4 years after baseline. Logistic regression was used to estimate the association between frailty and developing CKD.

**Results:**

A total of 3597 participants (mean age: 59.08 ± 8.94 years old, male: 49.9%) from CHARLS were included. Participants with pre-frailty or frailty status had a higher risk of developing CKD compared with robust participants (pre-frail vs robust, OR 1.78, 95% CI 1.37–2.32, *p* < 0.001; frail vs robust, OR 2.52, 95% CI 1.67–3.79, *p* < 0.001). Participants who had a robust status in the two surveys had a significantly lower risk of developing CKD (OR 0.51, 95% CI 0.36–0.75, *p* < 0.001) compared with those who never had a robust status.

**Conclusion:**

Frailty status is significantly associated with the incidence of CKD. The risk of CKD was lower in those who ever had a robust status than in participants who never had a robust status.

**Supplementary Information:**

The online version contains supplementary material available at 10.1007/s41999-024-01148-x.

## Key summary points

**Aim:**

Frailty is an important risk factor for a wide range of chronic diseases and for mortality risk. This study aims to explore the relationship between frailty and incidence of chronic kidney disease (CKD), particularly on the change and accumulation of frailty.

**Findings:**

An elevated risk of CKD was observed in pre-frail and frail participants at baseline compared with baseline robust participants. In addition, in both surveys, participants in the robust status one or more times had a significantly lower risk of CKD than those who had never had the robust status.

**Message:**

Frailty status is significantly associated with the risk of developing CKD.

**Supplementary Information:**

The online version contains supplementary material available at 10.1007/s41999-024-01148-x.

## Background

According to the National Bureau of Statistics of China, as of 2020, the number of persons 60 years and older in China will exceed 250 million, accounting for about 18.7 percent of the total population. Frailty is a common geriatric syndrome, in which adverse outcomes likely occur [1, 2]. A large body of research has demonstrated that frailty increases the risk of various age-related diseases (e.g., falls, disability, developing cardiovascular disease and mortality) [3–5]. Incoming studies have found that different changes in frailty status are also important: worsening frailty increases the risk of cardiovascular disease, whereas recovery from frailty decreases the risk of cardiovascular disease [6]. The frailty index (FI), which is derived by counting health deficits, is a reliable method for operationalizing frailty [7, 8].

Previous studies have found a relationship between chronic kidney disease (CKD) and frailty. In addition, frailty is highly prevalent in patients with CKD and is associated with increased mortality and risk of developing end-stage kidney disease in patients with CKD [9, 10]. Frailty is a reversible status [11, 12]. However, few studies have examined the relationship among frailty status, change or accumulation of frailty status, and developing renal disease.

The China Health and Retirement Longitudinal Study (CHARLS) is a large, interdisciplinary program of investigation that collects a set of high-quality microdata on households and individuals representing middle-aged and older people in China aged 45 years and over, which can be used to advance interdisciplinary research on aging. The present study aimed to find the relationship between frailty status, changes or accumulation of frailty, and the incidence of renal disease through the CHARLS.

## Participants and methods

### Study design and participants

In this study, CHARLS Wave 1 (2011) was considered as the baseline and Wave 3 (2015) was considered as the second survey (as only the two Waves included blood indicator data). Data from the baseline and second survey were used to assess the dynamics of frailty status and accumulation. A detailed description of CHARLS is shown in previously published literature and the official CHARLS website (http://charls.pku.edu.cn/) [13]. CHARLS was approved by the Ethical Review Committee of Peking University, and informed consent was obtained from each participant.

**Fig. 1:**
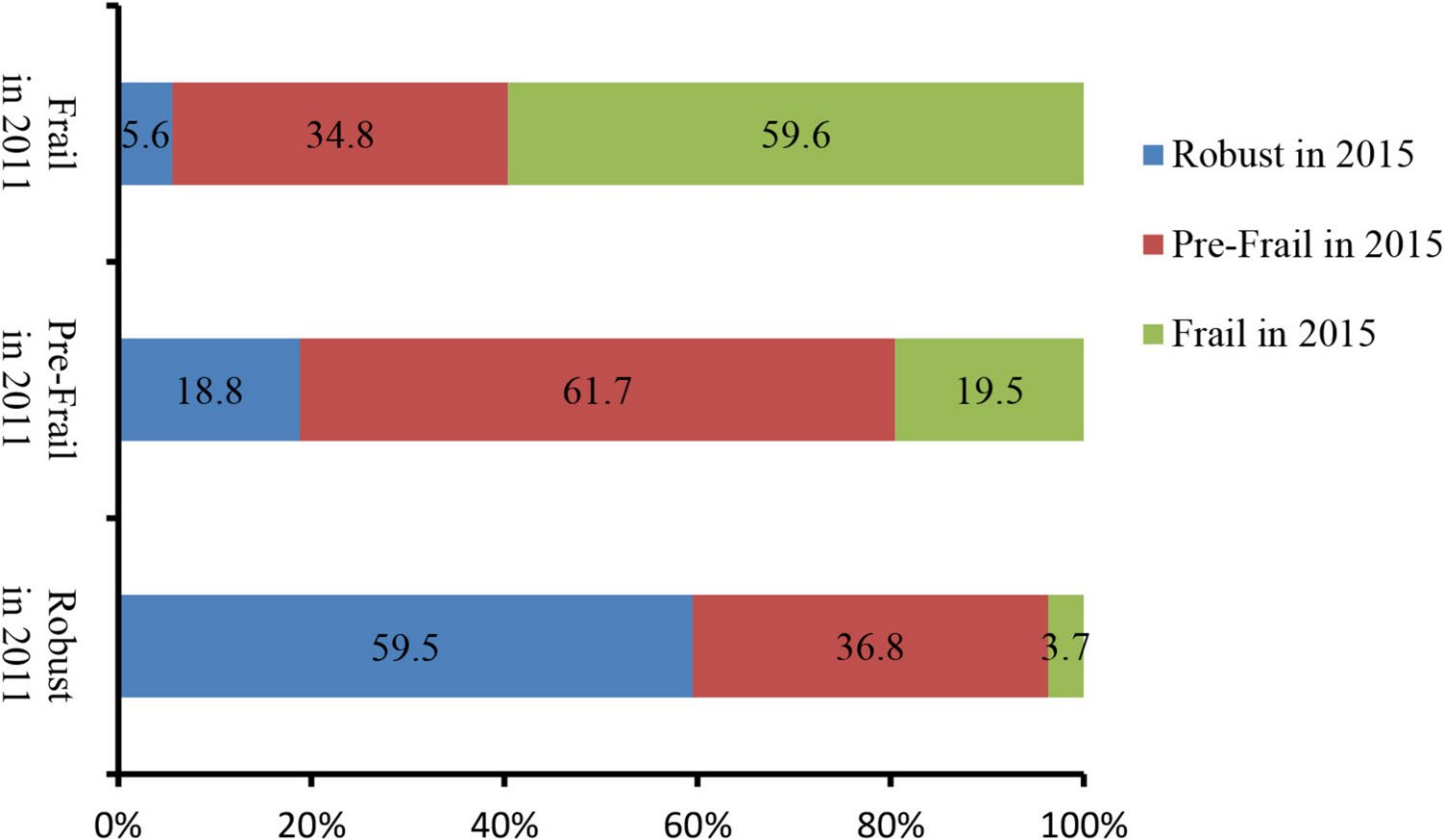
Number and proportion of participants with relative frailty status in 2011 and 2015

S-Fig. 1 shows the screening process for the study population. In analyzing baseline frailty, we initially excluded 5185 participants with missing FI calculation data at baseline based on FI calculation correlation entries and those younger than 45 years of age; then, we excluded 8814 participants with missing estimated glomerular filtration rate (eGFR) calculation data at baseline, participants with pre-existing nephropathy at baseline, and participants diagnosed with renal insufficiency based on eGFR calculation at baseline. A total of 3597 eligible participants were included. For analyses related to the change or accumulation of frailty, additional 1046 participants from the baseline sample who were missing data on FI calculations in 2015, participants who were missing data on eGFR calculations in 2015, participants who already had renal disease in 2013 and 2015 and participants who were diagnosed with renal insufficiency based on eGFR calculation in 2015 were excluded. Finally, a total of 2551 eligible participants were included.

As recommended by the Expert Consensus on the Diagnosis and Management of Chronic Kidney Disease in the Elderly (2018), the eGFR calculation in the present study was based on the CKD-EPIcr-cyst formula [14]. Creatinine was tested using the rate-blanked and compensated Jaffe creatinine method, and cystatin C was tested using the particle-enhanced turbidimetric assay in CHARLS. In addition, based on the studies from Chinese populations on age- and sex-related GFR thresholds among healthy populations, the low reference limit for eGFR was < 60 mL/min/1.73 m^2^ for ages ≥ 40 or 45 mL/min/1.73 m^2^ for ages ≥ 65 years. Participants who met these criteria in 2011 and 2015 were diagnosed of renal disease [15].

### FI

**Fig. 2:**
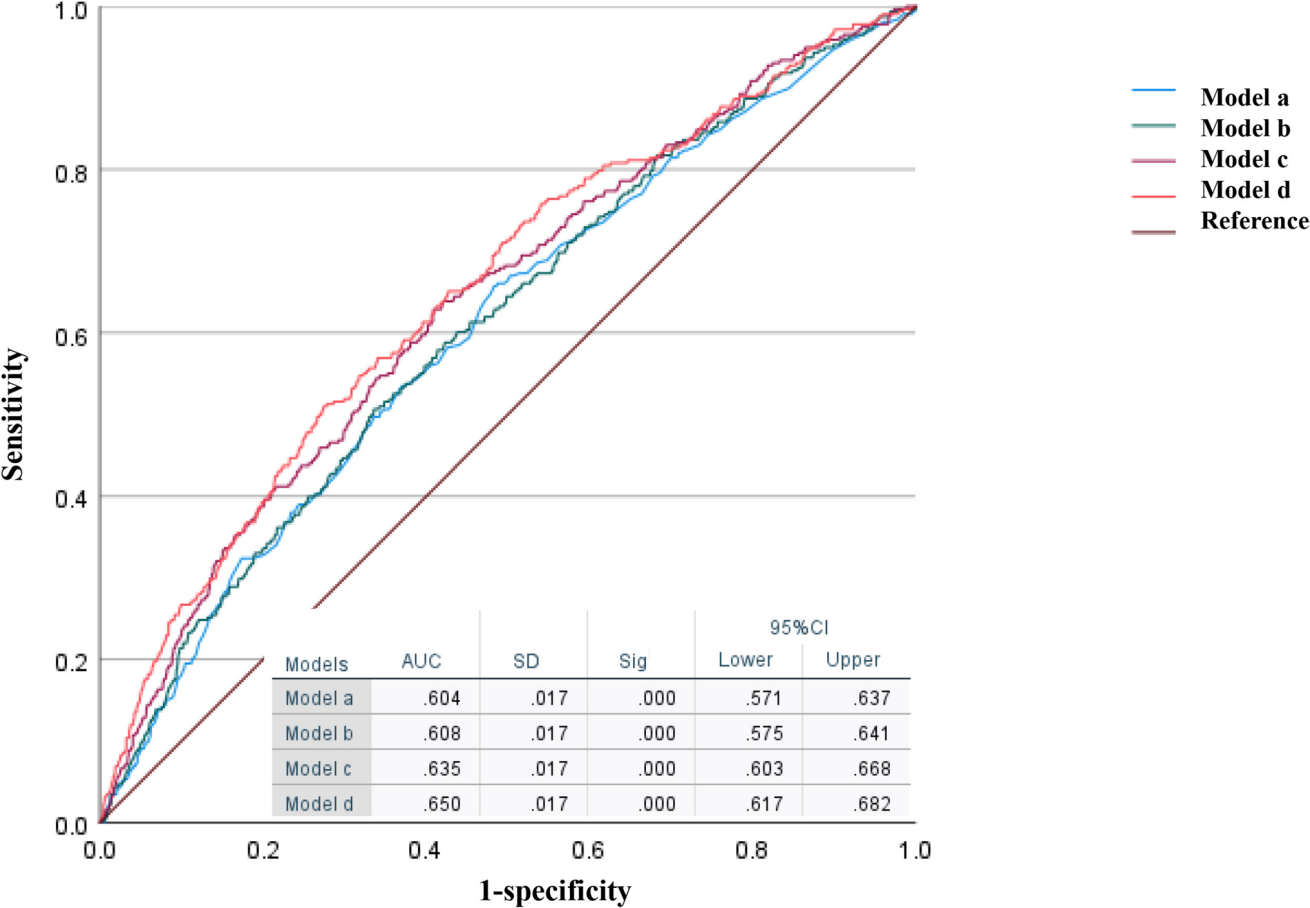
The receiver operating characteristic curves (ROC)s of 4 Models assessing the predictive ability of the frailty index for the incidence of CKD. (Notes: Model a was adjusted for age and sex; Model b was adjusted for age, sex, marital status, education, smoking status, drinking status, body mass index (BMI), glycated hemoglobin (HbA1c), systolic blood pressure (SBP), low-density lipoprotein cholesterol (LDL-C), eGFR and C-reactive protein; Model c was adjusted for age, sex, marital status, education, smoking status, drinking status, BMI, HbA1c, SBP, LDL-C, eGFR, C-reactive protein, diabetes and hypertension; Model d was adjusted for age, sex, marital status, education, smoking status, drinking status, BMI, HbA1c, SBP, LDL-C, eGFR, C-reactive protein, diabetes and hypertension, diabetes treatment (including taking at least one of Chinese traditional, Western modern medicine or taking insulin injections) and hypertension treatment (including taking Chinese traditional or Western modern medicine or both).)

In the present study, FI was constructed cumulatively for various age-related health deficits based on previous studies [6, 8]. A total of 31 items were selected to construct FI based on the CHARLS data (disability, physical functioning, cognition, and diseases; S-Table 1). Items 1–30 were dichotomized as 1 (with) or 0 (without). Item 31 (cognitive) is a continuous variable ranging from 0 to 1. The higher the cognitive scores, the poorer the cognitive ability is. Cognitive calculations were performed on the basis of CHARLS user guidance document and previous references [16, 17]. FI for each participant was calculated by dividing the sum of current health deficits by 31 (ranging from 0 to 1). The higher the FI value, the greater the frailty is. The density plots of FI at the 2011 and 2015 examinations are shown in S-Fig. 2. Frailty states were classified into three categories: robust (FI ≤ 0.10), pre-frailty (0.10 < FI < 0.25) and frailty (FI ≥ 0.25) [6, 18, 19]. Changes in frailty status were assessed on the basis of the frailty status reported in 2011 and 2015. The cumulative effect of frailty was also assessed using frailty status accumulation (the combination of two frailty statuses was categorized as ever robust and other).

### Follow-up and outcomes

The CHARLS was used to collect information on the occurrence of 14 common chronic diseases, including kidney disease. A kidney disease is defined as any condition other than kidney tumor or cancer [20]. The primary outcome of this study was developing CKD (except for tumors or cancer). In CHARLS, chronic diseases were determined by self-report or diagnosis by a physician. The follow-up endpoint was developing CKD (except for tumor or cancer; the endpoint event in 2015 also included people with renal insufficiency diagnosed by eGFR). In the second and subsequent surveys, developing CKD (except for tumor or cancer) was determined on the basis of a combination of several variables (whether or not one had kidney disease; self-knowledge or doctor’s notification) and the time of disease onset (whether or not one had kidney disease at the time of the last survey and the time of disease onset, whether or not the results of the last survey were correct and whether or not one developing CKD since the last survey and the time of disease onset).

When studying the relationship between baseline FI and developing CKD, the CHARLS second (2013), third (2015), fourth (2018) and fifth (2020) follow-up surveys were applied for tracking outcomes. When studying the relationship between the change or accumulation of frailty and the developing CKD, the CHARLS fourth (2018) and fifth (2020) follow-up surveys were applied for tracking the results.

### Statistical analysis

Statistical analyses were performed using SPSS 18.0 (SPSS Inc, Chicago, IL) and R software (version 4.3.2, R Foundation for Statistical Computing, Austria). Data were presented as number (percentage) for categorical variables and mean (SD) or median (interquartile range (IQR)) for continuous variables. Variables of interest were compared using one-way ANOVA, non-parametric tests, Chi-square tests, or Fisher exact tests as appropriate. Binary logistic regression analysis was used calculate the Odds Ratio (OR) and 95% confidence interval (CI) for the comparisons of clinical outcomes (PS: for those with developing CKD in 2013–2018, and data on time of disease onset were missing for many participants; and the data on time of disease onset were missing for participants diagnosed with renal insufficiency via eGFR calculation in 2015. In addition, the survey in 2020 did not provide data on the time of disease onset (S-Table 2). Based on the abovementioned results, COX risk regression models were not used in our analyses, but rather binary logistic regression models. The following multifactor binary regression models were used in this study: Model a was adjusted for age and sex. Model b was adjusted for age, sex, marital status, education, smoking status, drinking status, body mass index (BMI), glycated hemoglobin (HbA1c), systolic blood pressure (SBP), low-density lipoprotein cholesterol (LDL-C), eGFR and C-reactive protein. Model c was adjusted for age, sex, marital status, education, smoking status, drinking status, BMI, HbA1c, SBP, LDL-C, eGFR, C-reactive protein, diabetes and hypertension. Model d was adjusted for age, sex, marital status, education, smoking status, drinking status, BMI, HbA1c, SBP, LDL-C, eGFR, C-reactive protein, diabetes and hypertension, diabetes treatment (including taking at least one of Chinese traditional, Western modern medicine or taking insulin injections) and hypertension treatment (including taking Chinese traditional or Western modern medicine or both). Considering that the covariates were all missing at a rate of less than 10%, no treatment was applied to the missing values. *p* < 0.05 was considered statistically significant.

## Results

### Basic characteristics of all patients

The baseline frailty status analysis included 3597 participants (mean age: 59.08 ± 8.94 years, male (49.9%)). As shown in Table 1, frail participants were older, more likely to be female, and less likely to be married or with a partner than robust people. Frail participants also had a lower education level, and they smoked and consumed alcohol more than robust participants. In addition, frail participants had higher levels of SBP, HbA1c and CRP, as well as a lower level of eGFR. No statistically significant differences in BMI and LDL-C levels were found between the two groups. A total of 2551 participants (mean age: 58.42 ± 8.73 years, male (52.9%)) were included to analyze the change and accumulation of frailty. As shown in S-Table 3, the results were similar to those shown in Table 1. The number and proportion of participants with relative frailty status in 2011 and 2015 are shown in Fig. 1 and S-Table 4. Among the robust individuals at baseline, most of them (*n* = 809, 59.5%) remained robust after a 4-year follow-up (in 2015), 36.8% (*n* = 500) became pre-frail and 3.7% (*n* = 50) were classified as frail in 2015. On the contrary, of the individuals who were frail at baseline, 59.6% (*n* = 96) remained frail, 34.8% (*n* = 56) became pre-frail and nine (5.6%) were considered as robust at follow-up.

**Table 1:** Basic characteristics for baseline frailty

Variable	Robust (*N* = 1796)	Pre-frail (*N* = 1516)	Frail (*N* = 285)	*p* value
Frailty index, median (IQR)	0.05 (0.05–0.08)	0.148 (0.12–0.18)	0.34 (0.28–0.40)	< 0.001
Age, years	57.7 (8.8)	60.1 (8.90)	62.7 (8.5)	< 0.001
Female, *n* (%)	805 (44.8)	818 (54.0)	180 (63.2)	< 0.001
Married or partnered, *n* (%)	1638 (91.2)	1322 (87.2)	249 (87.4)	0.001
Education, *n* (%)				< 0.001
Below high school	1529 (85.2)	1384 (91.3)	269 (94.4)	
High or vocational school	222 (12.4)	112 (7.4)	12 (4.2)	
College or above	43 (2.4)	20 (1.3)	4 (1.4)	
Ever smoking, *n* (%)	785 (43.7)	582 (38.4)	100 (35.1)	0.001
Drinking, *n* (%)				0.008
Ever drinking more than once a month	659 (36.7)	472 (31.1)	85 (29.8)	
Ever drinking but less than once a month	170 (9.5)	151 (10.0)	29 (10.2)	
Never	967 (53.8)	893 (58.9)	171 (60.0)	
Diabetes, *n* (%)	26 (1.4)	131 (8.6)	47 (16.5)	< 0.001
Hypertension, *n* (%)	307 (17.1)	475 (31.3)	124 (43.5)	< 0.001
Treatment for diabetes, *n* (%)	18 (1.0)	89 (5.9)	31 (10.9)	< 0.001
Treatment for hypertension, *n* (%)	221 (12.3)	376 (24.8)	96 (33.7)	< 0.001
BMI, kg/m^2^	23.9 (13.4)	24.4 (18.0)	23.8 (3.7)	0.555
SBP, mmHg	128.70 (20.25)	131.18 (22.18)	134.86 (22.64)	< 0.001
eGFR, ml/(min·1.73 m^2^)	88.8 (78.1–99.9)	85.9 (74.7–96.1)	83.7 (72.0–93.9)	< 0.001
HbA1c, %	5.2 (0.7)	5.3 (0.8)	5.5 (1.0)	< 0.001
LDL-C, mg/dl	115.7 (35.2)	117.0 (34.2)	117.1 (34.5)	0.551
CRP, median (IQR), mg/L	0.9 (0.521.9)	1.1 (0.6–2.2)	1.1 (0.6–2.2)	0.004

### Follow-up data and clinical events

Table 2 demonstrates the number and proportion of outcome events during follow-up. The frail group suffered significantly more from CKD than the robust group (robust vs pre-frail vs frail: for baseline analyses, 125 [7.0] vs 191 [12.6] vs 45 [15.8], *p* < 0.001; for change and accumulation analyses, 59 [4.6] vs 86 [8.8] vs 19 [13.0], *p* < 0.001).

**Table 2:** Incidence rates of clinical events

Variable	Robust	Pre-frail	Frail	*p* value
CKD in baseline frailty status analyses, *n* (%)	125 (7.0)	191 (12.6)	45 (15.8)	< 0.001
CKD in change and accumulation of frailty analyses, *n* (%)	59 (4.6)	86 (8.8)	19 (13.0)	< 0.001

### Association between frailty and the incidence of CKD

First, we analyzed the correlation between FI and eGFR. Nonparametric simple correlation analysis indicated that baseline FI and total FI were significantly and negatively correlated with eGFR (S-Table 5).

To further explore the relationship between frailty and developing CKD, logistic regression analyses were performed. Table 3 shows the association between frailty status and developing CKD at baseline. We also plotted the receiver operating characteristic curves under different regression models with sequentially increasing area under the curve (Fig. 2). The closer the AUC is to 1.0, the more truthful the detection method. Model d has a maximum AUC of 0.650 and has the highest accuracy in predicting the occurrence of CKD. Unfortunately, none of the four models has an AUC of 0.8 or more (representing excellent predictive accuracy), but the P-value of the ROC curves for all models is < 0.001, which still makes sense. After adjusting for confounders, frail and pre-frail individuals had a higher risk of developing CKD than robust individuals (pre-frail vs robust, OR 1.78, 95% CI 1.37–2.32; frail vs robust, OR 2.52, 95% CI 1.67–3.79; both *p* < 0.001).

**Table 3:** Analysis of developing CKD by crude and adjusted odds ratios for frailty

Variable	For baseline frailty					
	Crude OR (95% Cl)	*p* value	Adjusted OR^a^ (95% Cl)	*p* value	Adjusted OR^b^ (95% Cl)	*p* value
Robust	Reference					
Pre-frail	1.92 (1.52–2.44)	< 0.001	1.96 (1.54–2.49)	< 0.001	1.90 (1.47–2.46)	< 0.001
Frail	2.51 (1.74–3.61)	< 0.001	2.58 (1.77–3.75)	< 0.001	2.82 (1.90–4.19)	< 0.001

			Adjusted OR^c^ (95% Cl)	*p* value	Adjusted OR^d^ (95% Cl)	*p* value
Robust			Reference			
Pre-frail			1.78 (1.36–2.32)	< 0.001	1.78 (1.37–2.32)	< 0.001
Frail			2.58 (1.68–3.80)	< 0.001	2.52 (1.67–3.79)	< 0.001

For frailty status						
	Crude OR (95% Cl)	*p* value	Adjusted OR^a^ (95% Cl)	*p* value	Adjusted OR^b^ (95% Cl)	*p* value
Frail or Pre-frail	Reference					
Ever robust	0.48 (0.35–0.65)	< 0.001	0.44 (0.31–0.61)	< 0.001	0.46 (0.32–0.66)	< 0.001

			Adjusted OR^c^ (95% Cl)	*p* value	Adjusted OR^d^ (95% Cl)	*p* value
Frail or Pre-frail			Reference			
Ever robust			0.52 (0.36–0.75)	< 0.001	0.51 (0.36–0.75)	< 0.001

*OR* Odds Ratio, *CI* confidence interval, *P* value compared with robust group for baseline frailty analysis and compared with frail of pre-frail group for frailty status analysis

^a^Odds ratios were multivariable-adjusted controlling for age and sex

^b^Odds ratios were multivariable-adjusted controlling for age, sex, marital status, education, smoking status, drinking status, BMI, SBP, HbA1c, eGFR, LDL-C and C-reactive protein

^c^Odds ratios were multivariable-adjusted controlling for age, sex, marital status, education, smoking status, drinking status, BMI, SBP, HbA1c, eGFR, LDL-C, C-reactive protein, diabetes, and hypertension

^d^Odds ratios were multivariable-adjusted controlling for age, sex, marital status, education, smoking status, drinking status, BMI, SBP, HbA1c, eGFR, LDL-C, C-reactive protein, diabetes, hypertension, treatment of diabetes, and treatment of hypertension

In frail cumulative analysis, participants who had a robust status in both frailty surveys had a significantly lower risk of developing CKD (OR 0.51, 95% CI 0.36–0.75, *p* < 0.001) than those who never had a robust status (Table 3). However, we did not see similar results in frailty change analyses, which may be related in part to the small sample of people included in this study, after rigorous exclusion (S-Table 6). Developing CKD was also analyzed by crude and adjusted ORs for frailty by tertiles and as a continuous variable, whose results were previously consistent (S-Table 7). In addition, after constructing a new FI (items 1–4 were removed), the abovementioned analysis was conducted again, and the results further support the previous findings (S-Table 8).

## Discussions

This study was the first to explore the association between frailty and the incidence of CKD with a high-quality retrospective investigation from China-CHARLS. An elevated risk of CKD was found in pre-frail and frail participants at baseline compared with baseline robust participants. In analyzing the accumulation of frailty (total FI), those with frail status had a higher risk of CKD than those with robust status. Moreover, we found that in both surveys (2011 and 2015), participants who had a robust status one or more times had a significantly lower risk of CKD than those who had never had the robust status. The abovementioned results, combined with the reversibility of frailty status, indicate that being robust has many benefits, which is similar to the benefits of physical exercise to the human body.

At present, China is facing a serious aging trend, and frailty is becoming an increasingly common condition of aging. Frailty is a multidimensional biopsychosocial syndrome, and previous studies have shown an association between frailty and various serious outcomes, such as falls, hospitalization, cardiovascular disease, and mortality risk [1, 22]. Most studies on frailty and kidney disease have focused on people who already have a kidney disease. For example, frail patients with CKD likely develop end-stage renal disease, and frail individuals with CKD have a higher risk of atherosclerotic events, heart failure events, and death [9, 10, 23]. Considerable clinical overlap is found between patients with kidney disease and those with frailty, and frailty is common in patients with CKD. However, whether chronic renal failure per se contributes to the incidence of frailty or whether frailty plays a role in the development of CKD remains unclear [24]. Several previous studies have explored the fact that renal insufficiency generally increases the risk of frailty [25–27]. Findings from CJASN found that rapid eGFR loss was associated with subsequent frailty (hazard ratio, 1.55; 95% CI 1.01–2.40). However, in the adjusted Cox model, baseline eGFR was found to be associated with frailty as a continuous and as a binary variable but not with the incidence of frailty [28]. In addition, few studies have been conducted on frailty and developing CKD. The results of this study indicated that pre-frail and frail participants have a higher risk of developing CKD than frail participants at baseline. The FI is a complex indicator that combines dozens of factors such as inflammation (significantly elevated CRP), age, and chronic disease, all of which indirectly explain and support the results of this study.

Apart from baseline frailty status, our study investigated the association of frailty accumulation with developing CKD, which has not been performed in previous studies. Some studies have found that frailty status is changeable, with some worsening and some improving [11, 12]. Our study found that the risk of developing CKD was significantly reduced, although robust was achieved only once in the two FI surveys. This finding highlights the importance of improving frailty and emphasizes the benefit (in this study, for developing CKD) of improving the frail status which is similar to the benefit of physical exercise to the human body.

This study has several strengths. First, to the best of our knowledge, this study is the first study to examine the association between frailty, frailty accumulation, frailty change and the incidence of CKD. Second, this study was conducted by applying a high-quality Chinese retrospective age-related cohort. CHARLS has a rigorous study design, and it is responsive to the Chinese population. Third, our study has some clinical implications: in conjunction with previous studies related to cardiovascular disease and mortality risk, incorporating frailty assessment into routine chronic disease assessment is necessary, not only in renal disease, especially in the older population and in those at high risk for renal disease or nephropathy. Individuals who are frail or pre-frail should be considered as an important target for the prevention of adverse events. The changes in frailty must also be monitored to measure the cumulative state of frailty and to provide guidance related to the reversal of frailty in high-risk populations.

This study also has some limitations. First, this study looked on frailty and developing CKD. Although kidney-related cancers were removed in the present study, kidney disease has several categories; thus, making a careful distinction is necessary. Second, most developing CKD in our study was based on self-reported information of physician diagnosis, which may lead to misclassification bias. Third, a large number of samples were deleted because of missing data related to baseline eGFR calculation and FI calculation, which resulted in only about 3000 individuals in this study. Fourth, according to KDIGO 2024 Clinical Practice Guidelines for Evaluation and Management of Chronic Kidney Disease, the diagnosis of CKD is decided by the presence of either of the following factors for a minimum of 3 months: decreased GFR or markers of kidney damage (albuminuria, urine sediment abnormalities, persistent haematuria, electrolyte and other abnormalities caused by tubular disorders, abnormalities detected by histology, structural abnormalities detected by imaging and history of kidney transplantation). However, CHARLS did not collect the markers of kidney damage. In the present study, newly diagnosed CKD included those identified by self-report or physician diagnosis as well as those diagnosed by eGFR, the latter of which was defined as CKD using only photostatic-corrected GFR. Fourth, for CKD diagnosis based on GFR, our study was age stratified concerning relevant studies in the Chinese population. When diagnosing chronic renal failure, the current guidelines do not include different eGFR values at different ages. In addition, although the models used in this study were adjusted for multiple confounders, residual confounders may still exist, such as physical activity (not taken because of missing data in CHARLS), diet, and sleep. Finally, only one cohort was applied in this study, which was validated only in the Chinese population. Furthermore, more real-world data and clinical trials are necessary to conduct further research.

## Conclusions

Frailty status is significantly associated with the incidence of CKD. Pre-frail and frail had a higher risk of CKD compared with the baseline robust. The risk of developing CKD was significantly lower in participants who had a robust status than those who never had a robust status. Therefore, delaying or reversing frailty status has important implications.

## Supplementary Information

Below is the link to the electronic supplementary material.

S-figure 1. Flow chart of the participants. Supplementary file1 (TIF 9626 kb)

S-figure 2. The density plots of FI at the 2011 examinations (A) in baseline frailty analysis and FI at the 2011 (B) and 2015 examinations (C) in change of frailty analysis. Supplementary file2 (TIF 19524 kb)

Supplementary file3 (DOCX 47 kb)

## Data Availability

Data supporting the results of this study are available from official websites http:// charls.pku.edu.cn and https://g2aging.org/.
