# Correction: Association of frailty index with incidence of chronic kidney disease: China Health and Retirement Longitudinal Study

**DOI:** 10.1007/s41999-025-01164-5

**Published:** 2025-03-17

**Authors:** Lisha Zhang, Yan Zhang, Yanru He, Fuxue Deng, Jiahong Xue

**Affiliations:** Department of Cardiovascular Medicine, The Second Affiliated Hospital, Xi’an Jiaotong University, Xi’an, 710004 Shaanxi People’s Republic of China

**Correction: European Geriatric Medicine** 10.1007/s41999-024-01148-x

The article Association of frailty index with incidence of chronic kidney disease: China Health and Retirement Longitudinal Study, written by Lisha Zhang · Yan Zhang · Yanru He · Fuxue Deng · Jiahong Xue, was originally published under exclusive license to The Author(s), under exclusive licence to European Geriatric Medicine Society. As a result of the subsequent decision to publish the article under the open access model, the article’s copyright notice was changed on 22 January 2025 to © The Author(s) 2025 and the article is now distributed under a Creative Commons Attribution [CC BY].

