# Supplementary figures and images for: Association of frailty index with incidence of chronic kidney disease: China Health and Retirement Longitudinal Study
Source: Eur Geriatr Med. 2025 Jan 15;16(2):681–8. doi: 10.1007/s41999-024-01148-x (PMC12014822; doi:10.1007/s41999-024-01148-x)

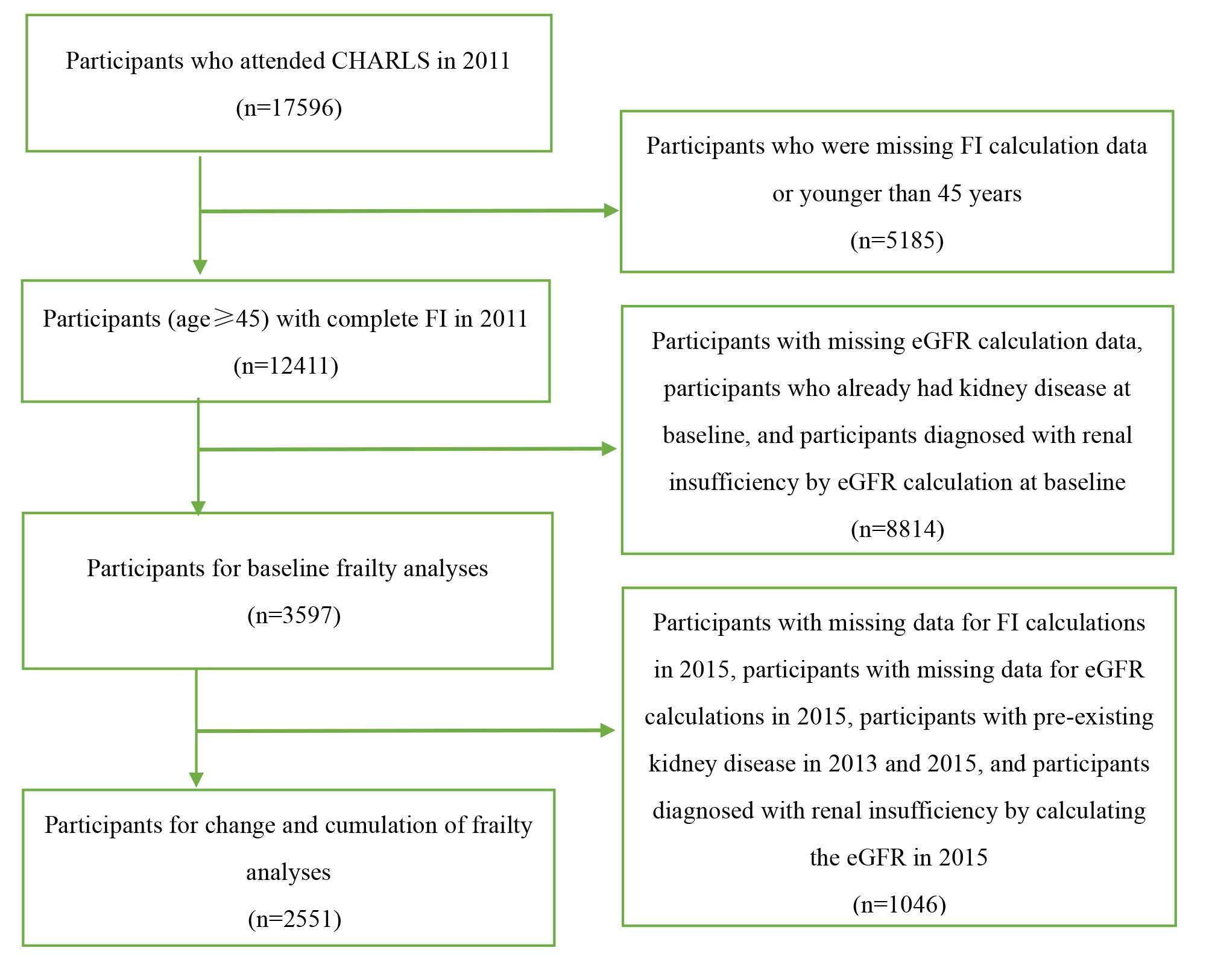

Supplement: Supplementary file 1 — S-figure 1. Flow chart of the participants. Supplementary file1 (TIF 9626 kb) [file 41999_2024_1148_MOESM1_ESM.tif]

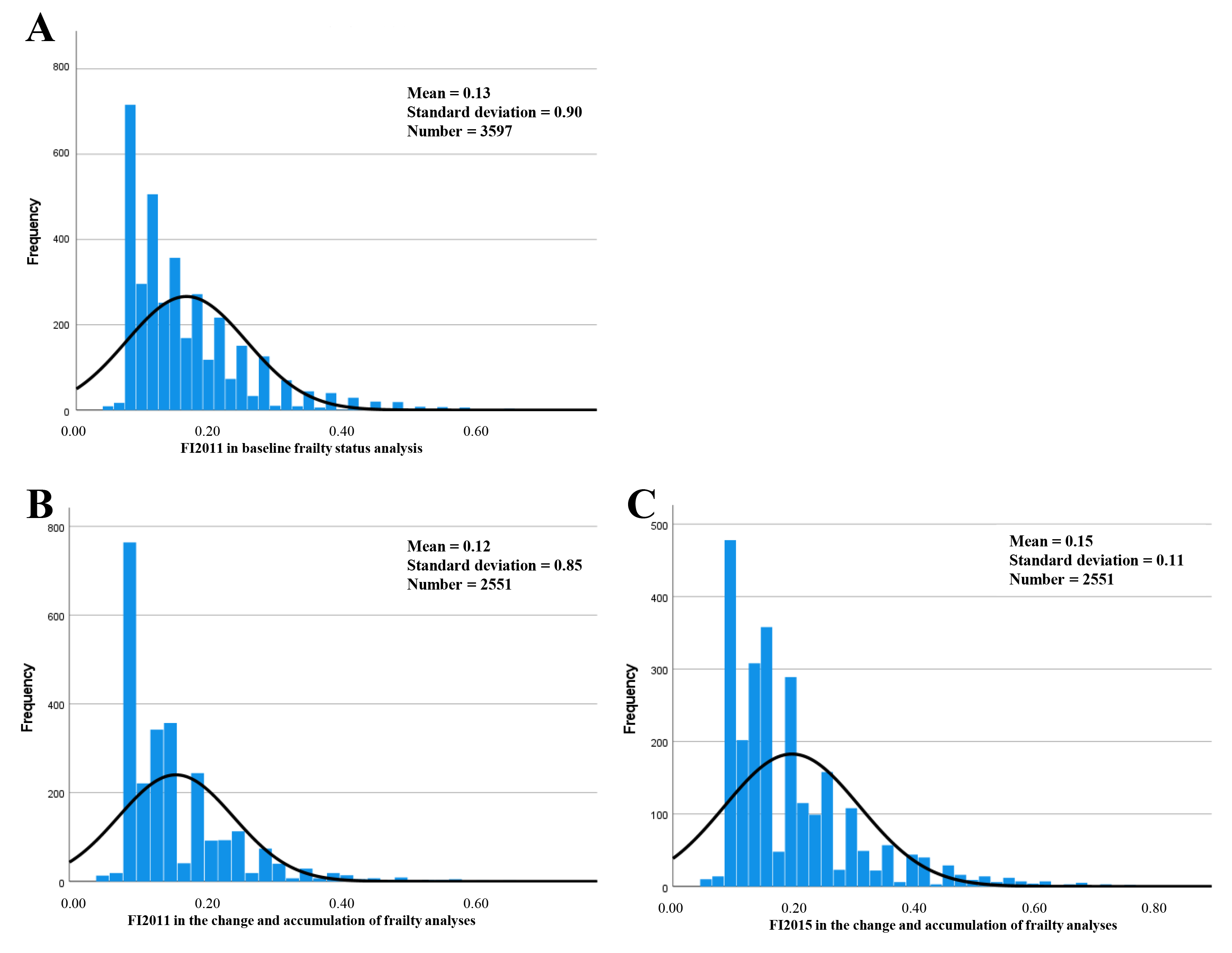

Supplement: Supplementary file 2 — S-figure 2. The density plots of FI at the 2011 examinations (A) in baseline frailty analysis and FI at the 2011 (B) and 2015 examinations (C) in change of frailty analysis. Supplementary file2 (TIF 19524 kb) [file 41999_2024_1148_MOESM2_ESM.tif]
