# Supplementary material for: Association of frailty index with incidence of chronic kidney disease: China Health and Retirement Longitudinal Study
Source: Eur Geriatr Med. 2025 Jan 15;16(2):681–8. doi: 10.1007/s41999-024-01148-x (PMC12014822; doi:10.1007/s41999-024-01148-x)
Supplement: Supplementary file 3 — Supplementary file3 (DOCX 47 kb) [file 41999_2024_1148_MOESM3_ESM.docx]

**Supplemental Table 1**. The 31 items used to construct the frailty index

| No | Description of the item | Cut-off value |
| --- | --- | --- |
| 1 | Self-reported physician diagnosed heart disease | Yes = 1, No = 0 |
| 2 | Self-reported physician diagnosed stroke | Yes = 1, No = 0 |
| 3 | Self-reported physician diagnosed hypertension | Yes = 1, No = 0 |
| 4 | Self-reported physician diagnosed diabetes | Yes = 1, No = 0 |
| 5 | Self-reported physician diagnosed cancer | Yes = 1, No = 0 |
| 6 | Self-reported physician diagnosed arthritis | Yes = 1, No = 0 |
| 7 | Self-reported physician diagnosed chronic lung disease | Yes = 1, No = 0 |
| 8 | Self-reported physician diagnosed any emotional, nervous, or psychiatric problems | Yes = 1, No = 0 |
| 9 | Self-reported physician diagnosed memory-related disease | Yes = 1, No = 0 |
| 10 | Self-reported vision problems | Yes = 1, No = 0 |
| 11 | Self-reported hearing problems | Yes = 1, No = 0 |
| 12 | Self-reported general health status | Very poor or poor = 1,  Very good, good, or fair = 0 |
| 13 | Difficulty with dressing | Yes = 1, No = 0 |
| 14 | Difficulty with bathing or showering | Yes = 1, No = 0 |
| 15 | Difficulty with eating | Yes = 1, No = 0 |
| 16 | Difficulty with getting in and out of bed | Yes = 1, No = 0 |
| 17 | Difficulty with using the toilet | Yes = 1, No = 0 |
| 18 | Difficulty with managing money | Yes = 1, No = 0 |
| 19 | Difficulty with taking medication | Yes = 1, No = 0 |
| 20 | Difficulty with shopping for groceries | Yes = 1, No = 0 |
| 21 | Difficulty with doing Household Chores |  |
| 22 | Difficulty with preparing meals | Yes = 1, No = 0 |
| 23 | Mobility: difficulty with walking 100 metres | Yes = 1, No = 0 |
| 24 | Mobility: difficulty with getting up from a chair after sitting for long periods | Yes = 1, No = 0 |
| 25 | Mobility: difficulty with climbing several flights of stairs without resting | Yes = 1, No = 0 |
| 26 | Mobility: difficulty with lifting or carrying weights over 10 pounds/jins | Yes = 1, No = 0 |
| 27 | Mobility: difficulty with picking up a coin from the table | Yes = 1, No = 0 |
| 28 | Mobility: difficulty with stooping, kneeling, or crouching | Yes = 1, No = 0 |
| 29 | Mobility: difficulty with reaching arms above shoulder level | Yes = 1, No = 0 |
| 30 | Depression: CESD-10 questionnaire | CESD-10 >10 = 1, ≤10 = 0 |
| 31 | Cognition: (memory test score (0-20) + orientation test score (0- 5) + 100-7 calculated test score (0-5) + Self-reported memory situation (0-5)) **/** 35 | Continuous variable, ranging  from 0 to 1 |

**Supplemental Table** **2**: Missing rates of CKD onset time.

| Variables | Number | Missing rate of onset time (%) |  |
| --- | --- | --- | --- |
| CKD in baseline frailty analyses | 278 |  |  |
| Onset time | 172 | 38.1 |  |
| CKD in change and accumulation of frailty analyses | 101 |  |  |
| Onset time | 65 | 35.6 |  |

**Supplemental Table** **3** Basic characteristics for frailty change or accumulation.

| Variable | Robust  N=1359 | Pre-Frail  N=1031 | Frail  N=161 | *P* value |
| --- | --- | --- | --- | --- |
| Frailty index, median (IQR) | 0.05(0.05-0.08) | 0.15(0.12-0.18) | 0.34(0.28-0.38) | <0.001 |
| Age, years | 57.4(8.6) | 59.4(8.8) | 61.3(8.0) | <0.001 |
| Female, n (%) | 560(41.2) | 541(52.5) | 101(62.7) | <0.001 |
| Married or partnered, n (%) | 1253(92.2) | 913(88.6) | 142(88.2) | 0.007 |
| Education, n (%) |  |  |  | <0.001 |
| Below high school | 1128(83.1) | 926(89.8) | 152(94.4) |  |
| High or Vocational school | 195(14.4) | 90(8.7) | 8(5.0) |  |
| College or above | 35(2.6) | 15(1.5) | 1(0.6) |  |
| Ever smoking, n (%) | 630(46.4) | 399(36.6) | 59(42.6) | <0.001 |
| Drinking, n (%) |  |  |  | 0.001 |
| Ever drinking more than once a month | 526(38.7) | 330(32.0) | 41(25.5) |  |
| Ever drinking but less than once a month | 140(10.3) | 111(10.8) | 21(13.0) |  |
| Never | 693(51.0) | 590(57.2) | 99(61.5) |  |
| Diabetes, n (%) | 21 (1.5) | 98 (9.5) | 26 (16.1) | <0.001 |
| Hypertension, n (%) | 235 (17.3) | 320 (31.0) | 74 (46.0) | <0.001 |
| Treatment for diabetes, n (%) | 16 (1.2) | 65 (6.3) | 18 (11.2) | <0.001 |
| Treatment for hypertension, n (%) | 165 (12.1) | 262 (25.4) | 60 (37.3) | <0.001 |
| BMI, kg/m2 | 23.6(3.5) | 24.8(21.6) | 24.1(3.7) | 0.136 |
| SBP, mmHg | 127.6(19.7) | 128.9(21.3) | 135.4(20.8) | <0.001 |
| eGFR, ml/(min·1.73 m^2^) | 89.3(79.0-100.1) | 86.8(75.8-97.3) | 85.2(73.8-94.8) | <0.001 |
| HbA1c, % | 5.2(0.6) | 5.3(0.8) | 5.2(0.7) | <0.001 |
| LDL-C, mg/dl | 115.0(34.5) | 116.2(33.3) | 117.4(34.1) | 0.559 |
| CRP, median (IQR),mg/L | 0.9(0.5-1.8) | 1.1(0.6-2.2) | 1.1(0.6-2.2) | 0.010 |

**Supplemental Table** **4** Change for frailty status.

| Variable | Robust2015, n | Pre-Frail2015, n | Frail2015, n | Total (%) |
| --- | --- | --- | --- | --- |
| Robust2011, n | 809 | 500 | 50 | 1359 (53.3) |
| Pre-Frail2011, n | 194 | 636 | 201 | 1031 (40.4) |
| Frail2011, n | 9 | 56 | 96 | 161 (6.3) |
| Total (%) | 1012 (39.7) | 1192 (46.7) | 347 (13.6) | 2551 (100) |

**Supplemental Table** **5** Simple correlation analysis between frailty index and eGFR in change or accumulation of frailty index analysis.

| Variable | GFR2011 | *P* value | GFR2015 | *P* value |
| --- | --- | --- | --- | --- |
| FI 2011 | -0.115 | <0.001 | -0.117 | <0.001 |
| FI 2015 | -0.179 | <0.001 | -0.172 | <0.001 |
| TFI | -0.165 | <0.001 | -0.157 | <0.001 |

FI frailty index, TFI total FI.

**Supplemental Table** **6** Analysis of developing CKD by crude and adjusted odds ratios for frailty change.

| Frail status in 2011 | Frail status in 2015 |  | | |  | | |  |  |
| --- | --- | --- | --- | --- | --- | --- | --- | --- | --- |
|  |  | Crude OR  (95% Cl) | *P* value | Adjusted OR^a^  (95% Cl) | | *P* value | Adjusted OR^b^  (95% Cl) | *P* value |  |
| Robust | Robust | reference |  |  | |  |  |  |  |
|  | Pre-frail | 1.46(0.86-2.48) | 0.158 | 1.50(0.87-2.58) | | 0.147 | 1.44(0.79-2.62) | 0.235 |  |
|  | Frail | 0.54(0.07-4.01) | 0.542 | 0.51(0.07-3.90) | | 0.519 | 0.48(0.06-3.86) | 0.487 |  |
| Pre-frail | Robust | reference |  |  | |  |  |  |  |
|  | Pre-frail | 1.15(0.61-2.18 | 0.660 | 1.29(0.68-2.45) | | 0.445 | 1.21(0.61-2.40) | 0.590 |  |
|  | Frail | 2.07(1.02-4.17) | 0.043 | 2.56(1.23-5.29) | | 0.012 | 2.23(1.00-4.98) | 0.050 |  |
| Frail | Robust | reference |  |  | |  |  |  |  |
|  | Pre-frail | 0.98(0.10-9.39) | 0.984 | 1.08(0.11-10.63) | | 0.950 | 1.08(0.10-12.10) | 0.953 |  |
|  | Frail | 1.09(0.12-9.67) | 0.938 | 1.30(0.14-12.18) | | 0.819 | 0.97(0.09-10.05) | 0.978 |  |
|  |  |  |  | Adjusted OR^c^  (95% Cl) | | *P* value | Adjusted OR^d^  (95% Cl) | *P* value |  |
| Robust | Robust |  |  | reference | |  |  |  |  |
|  | Pre-frail |  |  | 0.47 (0.06-3.81) | | 0.478 | 0.49 (0.06-3.94) | 0.499 |  |
|  | Frail |  |  | 0.34 (0.04-2.72) | | 0.307 | 0.35 (0.04-2.80) | 0.320 |  |
| Pre-frail | Robust |  |  | reference | |  |  |  |  |
|  | Pre-frail |  |  | 1.98 (0.87-4.50) | | 0.101 | 1.97 (0.87-4.46) | 0.107 |  |
|  | Frail |  |  | 1.77 (0.97-3.23) | | 0.062 | 1.76 (0.96-3.20) | 0.067 |  |
| Frail | Robust |  |  | reference | |  |  |  |  |
|  | Pre-frail |  |  | 0.98 (0.09-10.60) | | 0.988 | 1.05 (0.10-11.62) | 0.966 |  |
|  | Frail |  |  | 0.82 (0.23-2.90) | | 0.762 | 0.78 (0.21-2.85) | 0.708 |  |

OR Odds Ratio, CI confidence interval.

^a^ Odds ratios were multivariable-adjusted controlling for age and sex.

^b^ Odds ratios were multivariable-adjusted controlling for age, sex, marital status, education, smoking status, drinking status, BMI, SBP, HbA1c,eGFR, LDL-C, and C-reactive protein.

^c^ Odds ratios were multivariable-adjusted controlling for age, sex, marital status, education, smoking status, drinking status, BMI, SBP, HbA1c, eGFR, LDL-C, C-reactive protein, diabetes, and hypertension.

^d^ Odds ratios were multivariable-adjusted controlling for age, sex, marital status, education, smoking status, drinking status, BMI, SBP, HbA1c, eGFR, LDL-C, C-reactive protein, diabetes, hypertension, treatment of diabetes, and treatment of hypertension.

**Supplemental Table** **7** Analysis of developing CKD by crude and adjusted odds ratios for frailty by tertiles and as a continuous variable.

| Variable | For baseline frailty | | | | | | |
| --- | --- | --- | --- | --- | --- | --- | --- |
|  | Crude OR  (95% Cl) | *P* value | Adjusted OR^a^  (95% Cl) | *P* value | Adjusted OR^b^  (95% Cl) | *P* value |  |
| Lower third | reference |  |  |  |  |  |  |
| Middle third | 1.41(1.05-1.89) | 0.024 | 1.42(1.05-1.91) | 0.022 | 1.34(0.97-1.86) | 0.075 |  |
| Upper third | 2.20(1.68-2.90) | <0.001 | 2.27(1.71-3.01) | <0.001 | 2.31(1.70-3.14) | <0.001 |  |
| FI as a continuous variable | 20.67 (7.44-57.43) | <0.001 | 22.6 (7.87-65.11) | <0.001 | 22.21 (8.71-85.02) | <0.001 |  |
|  |  |  | Adjusted OR^c^  (95% Cl) | *P* value | Adjusted OR^d^  (95% Cl) | *P* value |  |
| Lower third |  |  | reference |  |  |  |  |
| Middle third |  |  | 1.27 (0.92-1.77) | 0.148 | 1.28 (0.92-1.78) | 0.138 |  |
| Upper third |  |  | 2.10 (1.53-2.88) | <0.001 | 2.10 (1.53-2.88) | <0.001 |  |
| FI as a continuous variable |  |  | 19.80 (6.07-64.63) | <0.001 | 19.35 (5.91-63.36) | <0.001 |  |

OR Odds Ratio, CI confidence interval, *P* value compared with the robust group.

^a^ Odds ratios were multivariable-adjusted controlling for age and sex.

^b^ Odds ratios were multivariable-adjusted controlling for age, sex, marital status, education, smoking status, drinking status, BMI, SBP, HbA1c, eGFR, LDL-C, and C-reactive protein.

^c^ Odds ratios were multivariable-adjusted controlling for age, sex, marital status, education, smoking status, drinking status, BMI, SBP, HbA1c, eGFR, LDL-C, C-reactive protein, diabetes, and hypertension.

^d^ Odds ratios were multivariable-adjusted controlling for age, sex, marital status, education, smoking status, drinking status, BMI, SBP, HbA1c, eGFR, LDL-C, C-reactive protein, diabetes, hypertension, treatment of diabetes, and treatment of hypertension.

**Supplemental Table** **8** Analysis of developing CKD by crude and adjusted odds ratios for new frailty^#^.

| Variable | | | For baseline frailty | | | | | | | | |
| --- | --- | --- | --- | --- | --- | --- | --- | --- | --- | --- | --- |
|  |  |  | Crude OR  (95% Cl) | | *P* value | Adjusted OR^a^  (95% Cl) | *P* value | Adjusted OR^b^  (95% Cl) | | *P* value |  |
| Lower third | | | reference | |  |  |  |  | |  |  |
| Middle third | | | 1.37 (1.02-1.83) | | 0.034 | 1.38 (1.03-1.85) | 0.032 | 1.41 (1.02-1.93) | | 0.036 |  |
| Upper third | | | 1.89 (1.43-2.48) | | <0.001 | 1.92 (1.45-2.54) | <0.001 | 2.04 (1.50-2.77) | | <0.001 |  |
| FI as a continuous variable | | | 8.67 (3.31-22.67) | | <0.001 | 8.90 (3.31-23.87) | <0.001 | 12.03 (4.18-34.62) | | <0.001 |  |
|  | | |  | |  | Adjusted OR^c^  (95% Cl) | *P* value | Adjusted OR^d^  (95% Cl) | | *P* value |  |
| Lower third | | |  | |  | reference |  |  | |  |  |
| Middle third | | |  | |  | 1.46 (1.06-2.01) | 0.020 | 1.45 (1.05-2.00) | | 0.024 |  |
| Upper third | | |  | |  | 2.08 (1.52-2.83) | <0.001 | 2.07 (1.52-2.82) | | <0.001 |  |
| FI as a continuous variable | | |  | |  | 12.60 (4.36-36.43) | <0.001 | 12.30 (4.24-35.67) | | <0.001 |  |
|  | | | Crude OR  (95% Cl) | | *P* value | Adjusted OR^a^  (95% Cl) | *P* value | Adjusted OR^b^  (95% Cl) | | *P* value |  |
| Robust | | | reference | |  |  |  |  | |  |  |
| Pre-frail | | | 1.78 (1.41-2.24) | | <0.001 | 1.80 (1.42-2.28) | <0.001 | 1.82 (1.41-2.35) | | <0.001 |  |
| Frail | | | 1.97 (1.38-2.81) | | <0.001 | 2.00 (1.39-2.88) | <0.001 | 2.19 (1.48-3.23) | | <0.001 |  |
|  | | |  | |  | Adjusted OR^c^  (95% Cl) | *P* value | Adjusted OR^d^  (95% Cl) | | *P* value |  |
| Robust | | |  | |  | reference |  |  | |  |  |
| Pre-frail | | |  | |  | 1.82 (1.41-2.36) | <0.001 | 1.82 (1.40-2.35) | | <0.001 |  |
| Frail | | |  | |  | 2.17 (1.47-3.21) | <0.001 | 2.16 (1.46-3.19) | | <0.001 |  |
| For frailty status | | | | | | | | | | |  |
|  | | | Crude OR  (95% Cl) | | *P* value | Adjusted OR^a^  (95% Cl) | *P* value | Adjusted OR^b^  (95% Cl) | | *P* value |  |
| Frail or Pre-frail | | | reference | |  |  |  |  | |  |  |
| Ever robust | | | 0.53 (0.39-0.73) | | <0.001 | 0.49 (0.35-0.68) | <0.001 | 0.47 (0.33-0.67) | | <0.001 |  |
|  | | |  | |  | Adjusted OR^c^  (95% Cl) | *P* value | Adjusted OR^d^  (95% Cl) | | *P* value |  |
| Frail or Pre-frail | | |  | |  | reference |  |  | |  |  |
| Ever robust | | |  | |  | 0.48 (0.34-0.69) | <0.001 | 0.48 (0.34-0.69) | | <0.001 |  |
| For frailty change | | | | | | | | | | | |
| Frail status in 2011 | | Frail status in 2015 | | Crude OR  (95% Cl) | *P* value | Adjusted OR^a^  (95% Cl) | *P* value | Adjusted OR^b^  (95% Cl) | | *P* value |  |
| Robust | Robust | | reference | |  |  |  |  | |  |  |
|  | Pre-frail | | 0.73 (0.42-1.28) | | 0.432 | 0.74 (0.41-1.31) | 0.296 | 0.83 (0.44-1.57) | | 0.571 |  |
|  | Frail | | 1.28 (0.50-3.32) | | 0.610 | 1.27 (0.48-3.34) | 0.635 | 1.54 (0.55-4.30) | | 0.413 |  |
| Pre-frail | Robust | | reference | |  |  |  |  | |  |  |
|  | Pre-frail | | 1.06 (0.58-1.91) | | 0.856 | 1.16 (0.63-2.11) | 0.636 | 1.26 (0.67-2.40) | | 0.476 |  |
|  | Frail | | 1.42 (0.73-2.78) | | 0.304 | 1.72 (0.86-3.45) | 0.126 | 1.39 (0.64-3.01) | | 0.405 |  |
| Frail | Robust | | reference | |  |  |  |  | |  |  |
|  | Pre-frail | | 2.15 (0.25-18.70) | | 0.487 | 2.15 (0.25-18.79) | 0.490 | 2.52 (0.26-24.18) | | 0.424 |  |
|  | Frail | | 1.18 (0.14-10.15) | | 0.881 | 1.24 (0.14-10.98) | 0.848 | 1.58 (0.16-15.28) | | 0.695 |  |
|  |  | |  | |  | Adjusted OR^c^  (95% Cl) | *P* value | Adjusted OR^d^  (95% Cl) | | *P* value |  |
| Robust | Robust | |  | |  | reference |  |  | |  |  |
|  | Pre-frail | |  | |  | 0.79 (0.42-1.50) | 0.473 | 0.79 (0.42-1.49) | | 0.459 |  |
|  | Frail | |  | |  | 1.45 (0.52-4.08) | 0.478 | 1.51 (0.53-4.26) | | 0.440 |  |
| Pre-frail | Robust | |  | |  | reference |  |  | |  |  |
|  | Pre-frail | |  | |  | 1.17 (0.60-2.22) | 0.643 | 1.20 (0.63-2.29) | | 0.582 |  |
|  | Frail | |  | |  | 1.19 (0.54-2.61) | 0.666 | 1.19 (0.54-2.61) | | 0.667 |  |
| Frail | Robust | |  | |  | reference |  |  | |  |  |
|  | Pre-frail | |  | |  | 3.37 (0.32-35.03) | 0.309 | 2.95 (0.29-29.96) | | 0.360 |  |
|  | Frail | |  | |  | 1.80 (0.17-18.98) | 0.624 | 1.71 (0.16-17.77) | 0.655 | |  |

^#^The FI was constructed by removing items 1-4 in S-table 1.

OR Odds Ratio, CI confidence interval, *P* value compared with the robust group.

^a^ Odds ratios were multivariable-adjusted controlling for age and sex.

^b^ Odds ratios were multivariable-adjusted controlling for age, sex, marital status, education, smoking status, drinking status, BMI, SBP, HbA1c, eGFR, LDL-C, and C-reactive protein.

^c^ Odds ratios were multivariable-adjusted controlling for age, sex, marital status, education, smoking status, drinking status, BMI, SBP, HbA1c, eGFR, LDL-C, C-reactive protein, diabetes, and hypertension.

^d^ Odds ratios were multivariable-adjusted controlling for age, sex, marital status, education, smoking status, drinking status, BMI, SBP, HbA1c, eGFR, LDL-C, C-reactive protein, diabetes, hypertension, treatment of diabetes, and treatment of hypertension.
